# The Effect of High and Variable Glucose on the Viability of Endothelial Cells Co-Cultured with Smooth Muscle Cells

**DOI:** 10.3390/ijms23126704

**Published:** 2022-06-16

**Authors:** Anna Ciechanowska, Ilona M. Gora, Stanislawa Sabalinska, Piotr Ladyzynski

**Affiliations:** Nalecz Institute of Biocybernetics and Biomedical Engineering, Polish Academy of Sciences, Ks. Trojdena 4, 02-109 Warsaw, Poland; igora@ibib.waw.pl (I.M.G.); ssabalinska@ibib.waw.pl (S.S.); pladyzynski@ibib.waw.pl (P.L.)

**Keywords:** diabetes, DM, diabetes complications, endothelial cells, smooth muscle cells, HUVECs, HUASMCs, co-culture, viability, high concentration of glucose, variable concentration of glucose, reactive oxygen species

## Abstract

Diabetes mellitus causes endothelial dysfunction. The aim of this study was to investigate the effect of normal (5 mmol/L), high (20 mmol/L), and fluctuating (5 and 20 mmol/L changed every day) glucose concentration in the culture medium on the viability of human umbilical vein endothelial cells (HUVECs) co-cultured with human umbilical artery smooth muscle cells (HUASMCs). The cultures were conducted on semi-permeable flat polysulfone (PSU) fibronectin-coated membranes immobilized in self-made inserts. The insert contained either HUVECs on a single membrane or HUASMCs and HUVECs on two membranes close to each other. Cultures were conducted for 7 or 14 days. Apoptosis, mitochondrial potential, and the production of reactive oxygen species and lactate by HUVECs were investigated. The results indicate that fluctuations in glucose concentration have a stronger negative effect on HUVECs viability than constant high glucose concentration. High and fluctuating glucose concentrations slow down cell proliferation compared to the culture carried out in the medium with normal glucose concentration. In conclusion, HUASMCs affect the viability of HUVECs when both types of cells are co-cultured in medium with normal or variable glucose concentration.

## 1. Introduction

Diabetes mellitus (DM) is a common disease in the world. In 2019, it affected 463 million adults aged 20 to 79 years [1]. It is a chronic disease characterized by hyperglycemia caused by a lack of insulin secretion from the pancreatic β cells (in type 1 diabetes—T1DM) or insulin resistance (in type 2 diabetes—T2DM). In people with diabetes, glucose levels can be consistently high, but in poorly controlled diabetes, it can also have high variability. Both of these conditions pose serious health risks [2].

Poorly controlled DM may cause acute metabolic life-threatening complications such as diabetic ketoacidosis (DKA) and hyperglycemic hyperosmolar state (HHS) [3,4,5,6]. Over years, DM leads to late complications, such as diabetic retinopathy, diabetic neuropathy, diabetic nephropathy, heart disease, and cerebrovascular disease. This is due to harmful effects of hyperglycemia, such as oxidative stress, which activates several pathways and processes accelerating endothelial dysfunction, which in turn leads to the development of diabetic antipathy [7,8,9]. Diabetes causes atherosclerosis and cardiovascular disease [10,11,12,13], which in turn cause endothelial dysfunction. Endothelial cells (ECs), as a monolayer, cover the inner part of the arteries and veins and have direct contact with blood [14]. Blood vessels, except for capillaries, consist of three layers: an outer layer—tunica adventitia, a middle layer—tunica media, and an inner layer, closest to the blood—tunica intima. The tunica adventitia is made of connective tissue; the tunica media consists of many layers of vascular smooth muscle cells (VSMCs), elastic fibers, and connective tissue; and the tunica intima consists of endothelium covered by a layer of connective tissue and internal elastic membrane which separates the tunica intima from the tunica media. Capillaries consist only of endothelium. Due to the number of cells that build endothelium in the human body, its mass, surface area, and the role it plays in many physiological processes, the endothelium has been recognized as an integral organ [15]. Endothelial cells control mass transport between tissues and blood, supplying water, nutrients, and hormones to the tissues surrounding the blood vessel and carrying waste products away. ECs produce vasoactive substances (endothelin, prostacyclin, endothelial-derived relaxing factor) and vasoconstrictors (thromboxane A2, angiotensin II, endothelin-1, prostaglandin H2) affecting smooth muscles. The basic function of smooth muscles is regulation of blood flow and pressure via vascular resistance [16]. Smooth muscle cells control the tone of the vessel wall. Proper communication between endothelial cells and smooth muscle cells is necessary to maintain homeostasis in the blood vessels [17,18,19]. The interaction between ECs and VSMCs to maintain vascular homeostasis occurs through extracellular matrix-mediated contact and through secreted molecules and extracellular vesicles (EVs) [20]. Heydarkhan-Hagvall et al. showed that co-cultures of ECs and VSMCs affect the gene expression of angiogenic factors [21]. In the event of endothelial damage, the interaction between the ECs and VSMCs is disturbed, leading to phenotypic and functional changes in VSMCs and inflammation that causes pathological remodeling of the vessel wall [22]. Due to the interaction between ECs and VSMCs, in vitro analysis of the pathophysiology of blood vessels applying co-cultures of both cell types should reflect in vivo conditions more closely than EC monocultures.

Human umbilical vein endothelial cells (HUVECs) are commonly used in vitro as a model to study the effects of different diseases and pathophysiological processes on the inner wall of blood vessels. For bioethical reasons, it is indicated to obtain human ECs for in vitro tests from the umbilical cord vein. After delivery, the umbilical cord is usually removed after umbilical cord blood is collected.

The effect of high glucose levels (which occur in people with diabetes) on in vitro cultured HUVECs has been fairly well researched.

It was demonstrated that elevated levels of glucose, mimicking the glucose levels found in people with diabetes, induce apoptosis of HUVECs; slow down proliferation; disrupt the cell cycle; induce DNA damage; decrease production of interleukin 8 (IL-8); increase the expression of inflammatory cytokine interleukin 6 (IL-6); decrease vascular endothelial growth factor (VEGF) levels; and decrease angiogenesis [23,24,25,26,27,28,29]. Several mechanisms and pathways have been identified that mediate apoptosis of HUVECs, including the formation of an excessive amount of reactive oxygen species (ROS) in mitochondria [30]; sequential activation of c-Jun NH(2)-terminal kinase and caspase-3 [31]; decreasing mitochondrial membrane potential and cellular ATP content; inhibition of fatty acid oxidation [32]; activation of Nuclear Factor-κB (NF-κB) and c-Jun NH2-terminal kinase [33]; activation of a phosphoinositide 3-kinase-regulated cyclooxygenase-2 pathway [34]; and suppression of hexokinase 2 expression in a mitochondrion-dependent manner [2].

The effect of glucose fluctuations on endothelial cells in vitro has been studied relatively little. Generally, all authors conclude that variable glucose concentration is much more detrimental to HUVECs than constant high glucose concentration [35,36,37,38,39,40,41,42,43,44,45]. There are no studies reporting on the effect of high or variable glucose levels in culture medium on HUVECs in co-cultures with human umbilical artery smooth muscle cells (HUASMCs). There are only few results concerning other kinds of human EC than HUVECs cultured in medium with high glucose concentration [46,47,48]. As was mentioned earlier, layers of VSMCs are separated in the blood vessel from endothelial cells by elastic fibers and connective tissue. During in vitro studies, in order to imitate the natural structure of the vascular wall, VSMCs should be separated from ECs with a semi-permeable layer. The co-cultures of endothelial cells with smooth muscle cells in a culture medium with a high glucose level were carried out in spheroids [46,48] and culture inserts (PICM03050; Millipore Corp., Bedford, MA, USA) [47].

Characterization and understanding of the mechanisms influencing the behavior of ECs co-cultured with SMCs in conditions simulating pathological blood glucose levels (not only constantly high but also fluctuating) may have a key impact on the development of new treatments for preventing endothelial dysfunction in people with diabetes. For this purpose, effects of all of the factors which interact with glucose concentration in influencing EC wellbeing should be identified, analyzed, and accounted for.

The aim of the current study was to investigate the effect of normal, high, and variable glucose concentrations in culture medium, mimicking the glucose levels found in people with diabetes, on the viability of HUVECs co-cultured with HUASMCs or cultured alone in vitro on semi-permeable flat polysulfone (PSU) membranes immobilized in self-made inserts. Such membranes, located close to each other, separate the HUVECs and HUASMCs from direct contact and simultaneously make possible the exchange of signaling molecules between them, bringing the culture conditions closer to the conditions in the human body.

## 2. Results

### 2.1. Apoptosis Analysis

**Figure 1:**
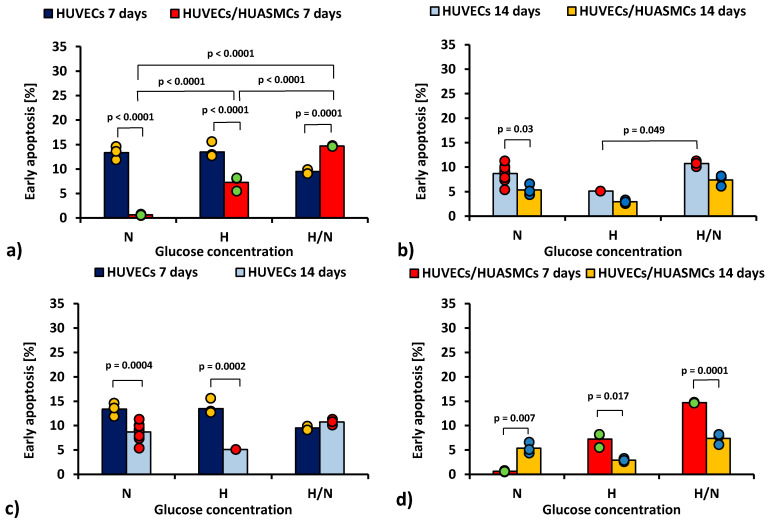
Percentage of early apoptotic HUVECs: after 7 days of culturing without HUASMCs or co-culturing with HUASMCs (**a**); after 14 days of culturing without HUASMCs or co-culturing with HUASMCs (**b**); after 7 and 14 days of culturing without HUASMCs (**c**); after 7 and 14 days of co-culturing with HUASMCs (**d**); in normal (N), high (H), or variable (H/N) glucose concentration on PSU flat membranes covered with fibronectin. The data are presented as scatter plot (yellow, green, red and blue dots) with corresponding bar graphs.

In Figure 1, the percentage of the early apoptotic (i.e., Annexin V-positive and 7AAD-negative) HUVECs is shown.

The ANOVA results show that all of the independent factors (glucose concentration, the time of culturing, and the presence or absence of HUASMCs as well as interactions of these factors in pairs and all three together) significantly influenced the percentage of HUVECs in the early apoptotic stage.

When HUVECs were co-cultured with HUASMCs for 7 days (Figure 1a), the percentage of early apoptotic HUVECs in medium with N and H glucose concentrations was lower (*p* < 0.0001) than in cultures without muscle cells (0.63% ± 0.73% vs. 13.4% ± 0.7% and 7.3% ± 0.7% vs. 13.5% ± 0.6%, respectively). Of note, only in H/N glucose concentrations was the percentage of early apoptotic HUVECs larger in co-cultures than in cultures without muscle cells (14.7% ± 0.9% vs. 9.5% ± 0.9%, *p* = 0.0001). In co-cultures, the percentage of early apoptotic HUVECs after 7 days was 11 times higher in medium with H glucose concentration than that with N glucose concentration (7.3% ± 0.7% vs. 0.63% ± 0.73%, *p* < 0.0001). However, in co-cultures with variable glucose concentration, the percentage of early apoptotic cells was more than two times higher (14.7% ± 0.9%) than in co-cultures with H glucose concentration (*p* < 0.0001) and 23 times higher than in co-cultures in N conditions (14.7% ± 0.9% vs. 0.63% ± 0.73%, *p* < 0.0001).

After 14 days (Figure 1b), the percentage of early apoptotic HUVECs cultured without HUASMCs in medium with N glucose concentration was higher than in co-cultures with HUASMCs (8.7% ± 0.4% vs. 5.4% ± 0.7%, *p* = 0.003). In medium with H/N glucose concentration, the percentage of early apoptotic cells was two times higher than in cultures in H conditions (10.7% ± 0.7% vs. 5.1% ± 1.3%, *p* = 0.049). Interestingly, in cultures of HUVECs without HUASMCs with N glucose concentration (Figure 1c), the percentage of early apoptotic cells was lower (*p* = 0.0004) after 14 days (8.7% ± 0.4%) than after 7 days (13.4% ± 0.7%). The same concerns cultures in medium with H glucose concentration (5.1% ± 1.3% vs. 13.5% ± 0.6%, *p* = 0.0002). When HUVECs were co-cultured with HUASMCs with H glucose concentration (Figure 1d), the percentage of early apoptotic cells was lower (*p* = 0.017) after 14 days (2.9% ± 0.7%) than after 7 days (7.3% ± 0.7%). The same concerns cultures in H/N medium (7.4% ± 0.7% vs. 14.7% ± 0.9%, *p* = 0.0001). In co-cultures in medium with N glucose concentration, this relationship was the opposite (i.e., 5.4% ± 0.7% vs. 0.63% ± 0.73%, *p* = 0.007).

In the analysis of the late apoptosis, after 14 days in medium with N glucose concentration the percentages of late apoptotic HUVECs was higher in co-cultures (10.8% ± 0.7% vs. 3.3% ± 0.4%, *p* < 0.0001). The percentage of apoptotic HUVECs co-cultured with HUASMCs in medium with H glucose concentration (6.2% ± 0.7%) was lower than in cultures in N glucose concentration (10.8% ± 0.7%, *p* = 0.01). The percentage of co-cultured HUVECs in the late apoptosis stage after 14 days in medium with N glucose concentration was higher than after 7 days (10.8% ± 0.7% vs. 4.1% ± 0.7%, *p* = 0.005).

**Figure 2:**
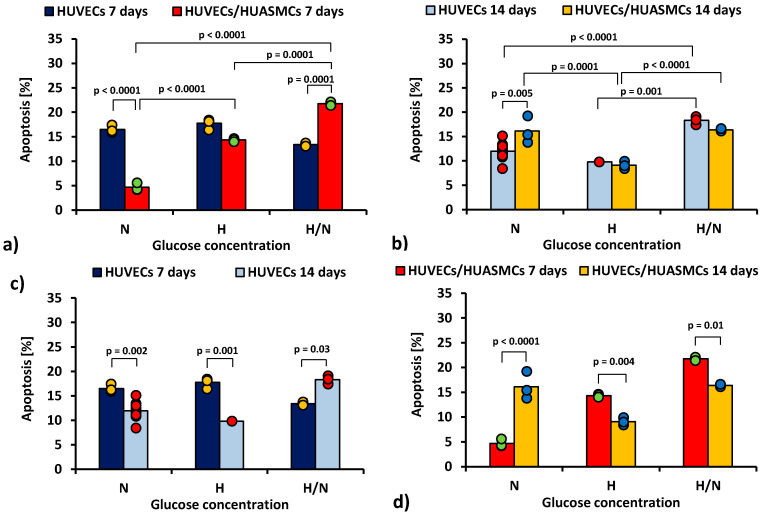
Total percentage of the early and late apoptotic HUVECs: after 7 days of culturing without HUASMCs or co-culturing with HUASMCs (**a**); after 14 days of culturing without HUASMCs or co-culturing with HUASMCs (**b**); after 7 and 14 days of culturing without HUASMCs (**c**); after 7 and 14 days of co-culturing with HUASMCs (**d**); in normal (N), high (H), or variable (H/N) glucose concentration on PSU flat membranes covered with fibronectin. The data are presented as scatter plot (yellow, green, red and blue dots) with corresponding bar graphs. The ANOVA results showed that all of the independent factors but the presence or absence of muscle cells, as well as all interactions of the independent factors in pairs and all three together, significantly influenced the percentage of HUVECs in early or late apoptosis.

In Figure 2, the total percentage of the early and late apoptotic HUVECs is shown.

After 7 days of culturing (Figure 2a) in medium with N glucose concentration, the percentage of apoptotic HUVECs cultured without smooth muscle cells was 3.5 times higher than co-cultures with HUASMCs (16.5% ± 0.8% vs. 4.7% ± 0.8%, *p* < 0.0001). In the cultures under H/N conditions, the relationship was the opposite (13.4% ± 1% vs. 21.8% ± 0.8%, *p* = 0.0001). In co-cultures in medium with N glucose concentration, the percentage of apoptotic HUVECs was three times lower (*p* < 0.0001) than in cultures in medium with H glucose concentration (4.4% ± 0.8% vs. 14.3% ± 0.8%) and almost five times lower (*p* < 0.0001) than in cultures in medium with H/N glucose concentration (21.8% ± 1.0%). The H/N glucose concentration was also significantly more harmful to HUVECs than H glucose concentration (*p* = 0.0001).

After 14 days (Figure 2b) in medium with N glucose concentration, the percentage of apoptotic HUVECs was lower when the HUVECs were cultured without muscle cells (12.0% ± 0.4% vs. 16.5% ± 1.0%, *p* = 0.005). In cultures of HUVECs, the percentage of apoptotic cells was higher when cells were cultured in H/N glucose concentration than in N (*p* < 0.0001) or H (*p* = 0.001) glucose concentration (18.3% ± 0.8% vs. 12.0% ± 0.4% vs. 9.8% ± 1.4%, respectively). The percentage of apoptotic HUVECs co-cultured with HUASMCs in medium with H glucose concentration (9.8% ± 1.4%) was lower than in cultures in H/N (16.4% ± 0.8%, *p* < 0.0001) or N glucose concentration (16.1% ± 0.8%, *p* = 0.0001).

The percentage of apoptotic HUVECs in cultures without HUASMCs in medium with N or H glucose concentration (Figure 2c) was lower (*p* = 0.002 and *p* = 0.001, respectively) after 14 days (12.0% ± 0.4% and 9.8% ± 1.4%, respectively) than after 7 days (16.5% ± 0.8% and 17.8% ± 1.4%, respectively). In HUVECs cultured in medium with H/N glucose concentration, this relationship was the opposite (i.e., 18.3% ± 0.8% vs. 13.4% ± 1.0%, *p* = 0.03).

In co-cultures in medium with H or H/N glucose concentration (Figure 2d), the percentage of apoptotic cells was lower (*p* = 0.004 and *p* = 0.01, respectively) after 14 days (9.1% ± 0.8% and 16.4% ± 0.8%, respectively) than after 7 days (14.3% ± 0.8% and 21.8% ± 1%, respectively). In medium with N glucose concentration, this relationship was the opposite (i.e., 16.1% ± 0.8% vs. 4.7% ± 0.8%, *p* < 0.0001).

### 2.2. Mitochondrial Membrane Potential Analysis

**Figure 3:**
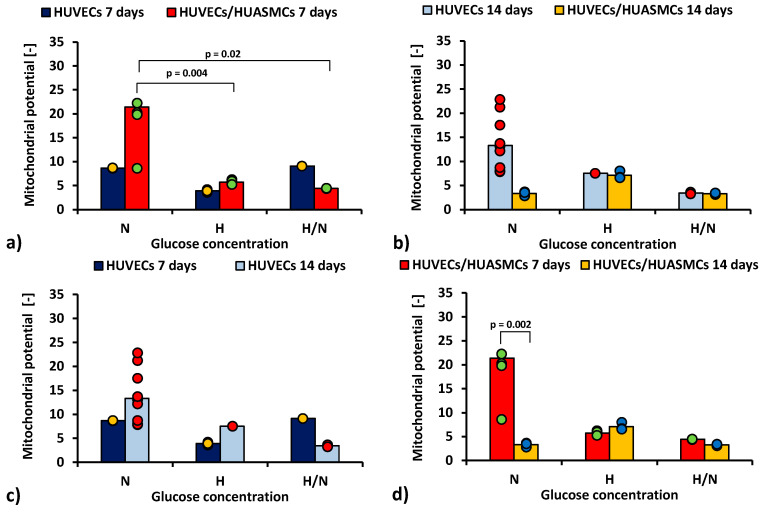
Mitochondrial membrane potential of HUVECs: after 7 days of culturing without HUASMCs or co-culturing with HUASMCs (**a**); after 14 days of culturing without HUASMCs or co-culturing with HUASMCs (**b**); after 7 and 14 days of culturing without HUASMCs (**c**); after 7 and 14 days of co-culturing with HUASMCs (**d**); in normal (N), high (H), or variable (H/N) glucose concentration on PSU flat membranes covered with fibronectin. The data are presented as scatter plot (yellow, green, red and blue dots) with corresponding bar graphs.

The ratio of red to green fluorescence signal in JC-1 cytometric analysis after 7 days of co-culturing HUVECs with HUASMCs in medium with N glucose concentration (Figure 3a) was nearly four times higher (*p* = 0.004) than in co-cultures with H glucose concentration (21.4 ± 2.2 vs. 5.71 ± 2.5) and five times higher (*p* = 0.02) than for cultures in H/N glucose concentration (21.4 ± 2.2 vs. 4.4 ± 3.5). The relationship between the mean values is inversely proportional to the relationship between the percentages of apoptotic cells presented in Figure 2a. However, the differences between means are small in comparison with SEMs, and, thus, they are mostly not significant. When the HUVECs were co-cultured with HUASMCs in N glucose concentration (Figure 3d), the mitochondrial membrane potential was six times lower after 14 days than after 7 days of culturing (3.3% ± 2.3% vs. 21.4% ± 2.2%, *p* = 0.002).

### 2.3. Intracellular Reactive Oxygen Species (ROS) Analysis

**Figure 4:**
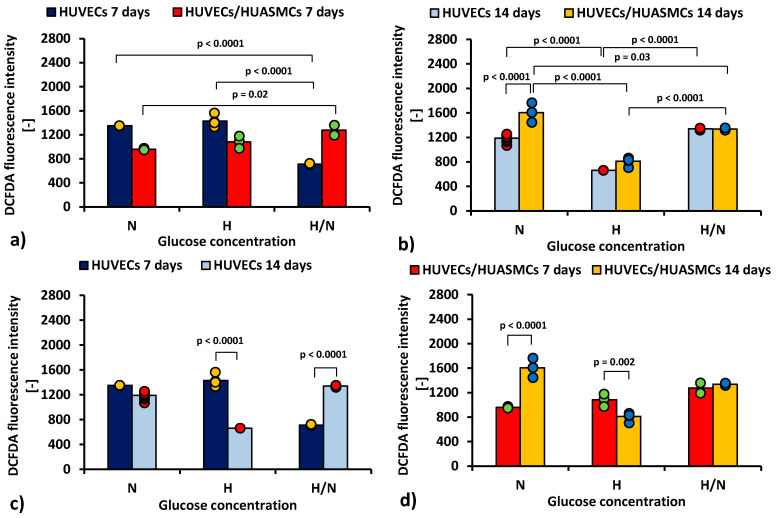
ROS production (DCFDA geometrical mean of the fluorescence intensity in cytometric analysis) by HUVECs: after 7 days of culturing without HUASMCs or co-culturing with HUASMCs (**a**); after 14 days of culturing without HUASMCs or co-culturing with HUASMCs (**b**); after 7 and 14 days of culturing without HUASMCs (**c**); after 7 and 14 days of co-culturing with HUASMCs (**d**); in normal (N), high (H), or variable (H/N) glucose concentration on PSU flat membranes covered with fibronectin. The data are presented as scatter plot (yellow, green, red and blue dots) with corresponding bar graphs.

Figure 4 shows cytometric analysis with DCFDA dye illustrating differences in production of ROS by HUVECs cultured under different conditions.

The results indicate that the mean ROS production by HUVECs after 7 days (Figure 4a) was about two times lower (*p* < 0.0001) in cultures with H/N glucose concentration (713 ± 53) than in those with N and H glucose concentrations (1350 ± 43 and 1428 ± 38, respectively). In co-cultures, the mean ROS production by HUVECs was lower in cultures with N glucose concentration than in cultures with H/N glucose concentration (961 ± 53 vs. 1276 ± 53, *p* = 0.002).

After 14 days, ROS production in HUVECs cultured without HUASMCs (Figure 4b) was lower (*p* < 0.0001) in cultures with H glucose concentration (662 ± 75) than in cultures with N and H/N glucose concentrations (1188 ± 24 and 1338 ± 43, respectively). In co-cultures in medium with N glucose concentration, the mean ROS production was higher (*p* < 0.0001) than in cultures of HUVECs without muscle cells (1606 ± 43 vs. 1188 ± 24). In HUVECs co-cultured with HUASMCs in medium with H glucose concentration, ROS production (810 ± 34) was lower (*p* < 0.0001) than in co-cultures in H/N and N glucose concentrations (1337 ± 53 and 1606 ± 43, respectively). The difference between production of ROS in HUVECs co-cultured in N and in H/N glucose conditions was also significant (1606 ± 43 vs. 1337 ± 53, *p* = 0.03).

The mean ROS production for HUVECs in cultures without HUASMCs in medium with H glucose concentration (Figure 4c) was lower after 14 days than after 7 days (662 ± 75 vs. 1428 ± 38, *p* < 0.0001). In HUVECs cultured in medium with H/N glucose concentration, this relationship was the opposite (1338 ± 43 vs. 713 ± 53, *p* < 0.0001). When HUVECs were co-cultured with HUASMCs in H glucose concentration (Figure 4d), the ROS production was lower after 14 days than after 7 days (810 ± 34 vs. 1051 ± 43, *p* = 0.002). In HUVECs co-cultured with HUASMCs in medium with N glucose concentration, this relationship was the opposite (1606 ± 43 vs. 961 ± 53, *p* < 0.0001).

### 2.4. Lactate Production by HUVECs

**Figure 5:**
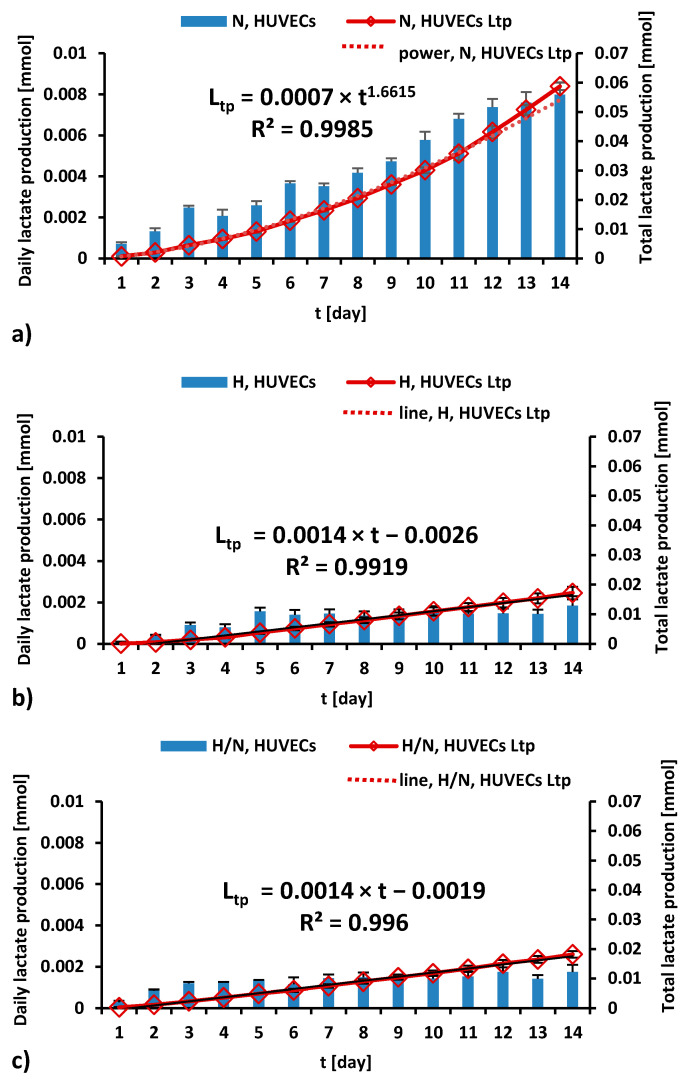
The daily (blue bars) and total (red line) lactate production during a 14-day culture of HUVECs in medium with N (**a**), H (**b**), and H/N (**c**) glucose concentration; L_tp_—total lactate production.

**Figure 6:**
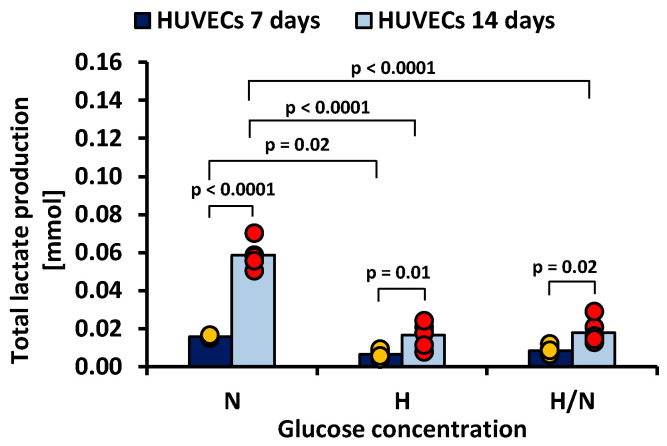
Lactate production by HUVECs after 7 and 14 days of culturing in medium with normal (N), high (H), or variable (H/N) glucose concentration on PSU flat membranes covered with fibronectin. The data are presented as scatter plot (yellow, green, red and blue dots) with corresponding bar graphs.

Lactate production by HUVECs cultured in culture medium with different glucose concentrations is presented in Figure 5 and Figure 6.

As can be seen in Figure 5a, during 14 days of culturing in medium with N glucose concentration, daily lactate production by HUVECs was higher every day, and the total lactate production was higher every day, in accordance with the power trend line.

For the cultures carried out in medium with H (Figure 5b) and H/N (Figure 5c) glucose concentrations, the daily production of lactate increases until the fifth or third day, respectively, and then it stabilizes. The trend lines for the total lactate production of HUVECs cultured in medium with H and H/N glucose concentrations are similar and take the form of a linear equation.

Figure 6 shows that, after 7 days, total lactate production was higher in cultures in medium with N glucose concentration than in that with H glucose concentration (0.0159 ± 0.0019 mmol vs. 0.0066 ± 0.0019 mmol, *p* = 0.02). After 14 days, the highest lactate production was also noted in the case of cultures in medium with N glucose concentration (0.0586 ± 0.0019 mmol), and it was significantly greater (*p* < 0.0001) than in cultures in medium with H or H/N glucose concentration (0.0166 ± 0.0019 mmol and 0.0179 ± 0.0019 mmol, respectively).

### 2.5. Summary of the Key Findings

- The relationship between the mean values of mitochondrial potential in HUVECs cultured alone and in the co-culture with HUASMCs is inversely proportional to that seen in the percentage of apoptotic cells and the production of ROS for all analyzed glucose concentrations in the medium.

#### 2.5.1. Influence of High and Fluctuating Glucose Concentrations on HUVECs

- After 7 days of co-culturing HUVECs with HUASMCs, the H/N glucose concentration was significantly more harmful to HUVECs than H glucose concentration.
- Regardless of whether HUVECs were cultured alone or in co-culture with HUASMC, after 14 days of culturing in medium with H/N glucose concentration, the percentage of apoptotic cells was higher than after culturing in medium with H glucose concentration. A similar relationship was observed in the case of ROS production.

#### 2.5.2. Influence of HUASMCs on HUVECs

- After 7 days of culturing, the percentage of HUVECs in total apoptosis was significantly lower for cells co-cultured with HUASMCs than those cultured alone in culture medium with N glucose concentrations, while it was significantly higher for HUVECs cultured in medium with H/N glucose concentration.
- After 14 days of culturing, the percentage of HUVECs cultured in N glucose concentration in total apoptosis was significantly higher for cells co-cultured with HUASMCs than for cells cultured without HUASMCs. A similar relationship was observed for ROS production.

#### 2.5.3. Lactate Production

- Daily lactate production by HUVECs cultured in culture medium with N glucose concentration was greater each day, and total lactate production was greater each day, following the power trend line. When HUVECs were cultured in medium with H and H/N glucose concentrations, the daily production of lactate increased until the fifth and third day, respectively, and then stabilized up to the 14th day of culture. The total lactate production followed a linear relationship with time.

## 3. Discussion

Our study was aimed at investigating the effect of normal, high, and variable glucose concentrations in culture medium (mimicking the glucose levels found in people with diabetes) on the viability of HUVECs co-cultured in vitro with HUASMCs on semi-permeable flat polysulfone membranes immobilized in self-made inserts. Cultures of HUVECs without HUASMCs were performed as reference. The cultures were conducted for 7 or 14 days. Cell viability was characterized by the percentage of early and late apoptotic cells, the total percentage of apoptotic cells, mitochondrial membrane potential, ROS production, and lactate production. According to the results of the literature review which we conducted, the impact of human smooth muscle cells on the viability of human endothelial cells co-cultured on PSU membranes in medium with different glucose concentrations has not been studied before.

Overall, depending on the viability parameter tested, results were influenced either by all three independent factors that were analyzed (i.e., presence or absence of the smooth muscle cells in culture, glucose concentration, and culture time) and their interactions or only by selected combinations of these factors.

The presence of muscle cells in culture (independently of other factors or in interaction with them) was found to have a significant effect on the parameters of cell viability, especially on the percentage of apoptotic cells and the production of ROS. The effect of HUASMCs on HUVEC viability was different depending on the glucose concentration in the culture medium and the culture time.

The results of the analysis of mitochondrial membrane potential were consistent with the results of the analysis of the percentage of apoptotic cells, although this was not always statistically significant. A faster pace of apoptosis corresponded to lower values of mitochondrial membrane potential and vice versa. Apoptosis is connected with depolarization of the membrane potential [49,50].

Excess ROS production in the mitochondria is one of the causes of accelerated apoptosis [51]. The results of the ROS production analysis indicate that for most combinations of independent variables, increased ROS production could be a factor leading to HUVECs apoptosis. ROS production corresponded with the results of the apoptosis analysis after 7 and 14 days of culturing in medium with different glucose concentrations, i.e., the level of ROS was higher when the corresponding percentage of apoptotic cells was higher.

We found a significant effect of HUASMCs on HUVECs apoptosis in 7-day-old cultures in medium with N and H/N glucose concentrations and in 14-day-old cultures in medium with N glucose concentration. It is noteworthy that in the 14-day cultures, the effect of HUASMCs on the apoptosis of HUVECs cultured in medium with N-glucose concentration was lesser than in the 7-day cultures (*p* < 0.0001 vs. *p* = 0.05). Our research on the influence of HUASMCs on the viability of HUVECs was aimed at showing that, when conducting tests on the influence of high and variable glucose concentration in the culture medium (simulating blood glucose levels in people with diabetes) on the viability of HUVECs, other factors should also be considered, such as, for example, the presence of HUASMCs in the culture that makes the conditions in vitro more similar to those in vivo. It was demonstrated [2] that the effect of glucose concentration on endothelial cell viability in vitro depended on the substrate which was used for cell culturing. The “gold standard” of the conditions under which in vitro tests should be carried out for the effect of high and fluctuating glucose levels on endothelial cell viability and behavior has not yet been defined, such that the results of the various tests cannot be compared. Defining such a “gold standard” seems to be crucial in conducting this type of research and, in the future, establishing the correct treatment and means of prevention of vascular complications in people with diabetes. We hope that the results of our research will contribute to the initiation of the definition of such a gold standard.

The results of our study show (Figure 2a,b) that the percentage of apoptotic HUVECs in co-cultures with HUASMCs after 7 and 14 days and in cultures of HUVECs without muscle cells after 14 days was higher if the glucose concentration was variable than if it was constantly high. This confirms the results of studies reported by others in tests carried out without HUASMCs in culture vessels, in which they showed that fluctuations of glucose concentration in culture medium, mimicking glucose fluctuations in people with diabetes, were more harmful to endothelial cells than the constant high concentration of glucose [35,36,37,38,39,40,41,42,43,44,45].

We observed no significant effect of glucose concentration on HUVEC apoptosis after 7 days in cultures without HUASMCs. The presence of HUASMCs in the culture, however, significantly reduced the apoptosis of HUVECs (especially early apoptosis) cultured in medium with normal and high glucose concentrations and increased the apoptosis of HUVECs cultured in variable glucose concentration.

**Figure 7:**
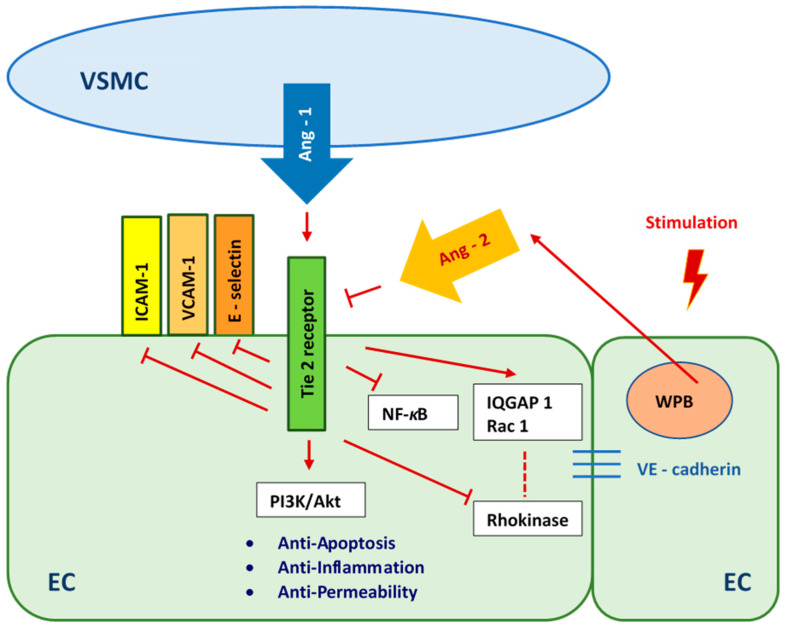
Angiopoietin 1/Tie 2 pathway as a reaction to high- and fluctuating-glucose stimulation. VSMC—Vascular Smooth Muscle Cells, ICAM-1 - Intercellular Adhesion Molecule 1, VCAM-1—Vascular Cell Adhesion Molecule 1, Ang-1—Angiopoietin 1, Ang-2—Angiopoietin 2, NF-κB—Nuclear Factor-κB, PI3K/Akt—the phosphatidylinositol 3-kinase/protein kinase B pathway, IQGAP1—IQ motif of the GTPase-1 activating protein 1, Rac 1—GTPase Rac1, EC—Endothelial Cell, WPB—Weibel-Palade Body.

Angiopoietin 1 (Ang 1) is secreted by vascular smooth muscle cells [52,53,54,55,56]. It is a ligand and strong agonist of the endothelial receptor tyrosine kinase 2 (tunica intima endothelial kinase 2—Tie 2). Angiopoietin 1 activates the Tie 2 receptor by inducing receptor tyrosine phosphorylation. When endothelial cells are stimulated, they release Angiopoietin 2 (Ang 2) from Weibel–Palade Bodies (WPB). As a result, the Ang 1/Tie 2 pathway propagates anti-inflammatory, anti-permeability, and anti-apoptotic signals [57,58,59]. We presume that abundant Ang 1 secreted by HUASMCs enhanced the viability of HUVECs which were cultured in medium with normal glucose concentration (Figure 7). The appearance of Ang 2 as a result of inflammation [55] induced by high and variable glucose concentrations caused, respectively, threefold and greater than fourfold higher apoptosis of HUVECs compared to the cultures carried out in medium with normal glucose concentration. The culture medium with variable glucose levels, after 7 days of co-culturing HUVECs with HUASMCs, is more toxic to HUVECs than the medium with persistently high glucose levels, due to the Ang 1/Tie 2 pathway and high Ang 2 expression. After 14 days of co-culture, the apoptosis of HUVECs cultured in medium with high and variable glucose levels decreased compared to the results after 7 days, which may indicate an enhancement of the protective mechanism of endothelial cells via the Ang 1/Tie 2 pathway. It may also indicate the adaptation of HUVECs to high and variable glucose levels by regulating glucose transport into the cell with the use of glucose transporters. Transport of glucose to the endothelial cells is insulin-independent and is mediated mainly by one type of transporter—GLUT1 [60,61]. It was shown that high glucose concentration in a short period of time causes high glucose uptake but decreases glucose transport over the longer term in endothelial cells [62,63]. High glucose concentration in the medium downregulates the GLUT1 transporter, and, consequently, glucose uptake by endothelial cells is also decreased [64].

The lowest percentage of apoptotic HUVECs was found in cultures in medium with normal glucose concentration. This result corresponded to the significantly higher mitochondrial membrane potential of these cells compared to the mitochondrial membrane potential of HUVECs cultured in medium with high or variable glucose concentration. However, there was no significant difference between HUVECs cultured in medium with normal and high glucose concentrations in terms of ROS production. In cultures in medium with variable glucose concentration, the production of ROS was only 1.3 times higher than that of cells cultured in medium with normal glucose concentration. When HUVECs were co-cultured with HUASMCs in normal glucose medium, we observed a protective effect of HUASMCs on HUVECs. This protective mechanism had less of an effect on HUVECs cultured in medium with high glucose concentration and had no effect on cells cultured under fluctuating glucose conditions. We hypothesize that the protective effect which we observed was due to the Angiopoietin 1/Tie 2 (Ang 1/Tie 2) pathway [52,53,54,55]. This pathway is critical for the prevention of cell death (Figure 7).

Another explanation for the apoptosis of HUVECs decreasing with the duration of the culture, but not in HUVECs cultured without HUASMCs in medium with variable glucose concentration or HUVECs co-cultured with HUASMCs in medium with normal glucose concentration, may be the activation of protective and repair mechanisms. Reactive oxygen species, which are formed in mitochondria as a result of hyperglycemia, damage endothelial cells; this, in turn, plays a key role in the pathogenesis of blood vessel disease [65,66,67,68]. Hyperglycemia in people with diabetes is associated with the activation of various ROS-producing pathways: hexosamine, polyol, methylglyoxal, and protein kinase C (PKC) [69,70]. All of these pathways lead to ROS production, which ultimately leads to inflammation. The overproduction of ROS by the mitochondria activates mechanisms that reduce their production. This must also be the case for the cell cultures that we carried out. The production of mitochondrial ROS damages DNA in mitochondria, which in turn activates repair enzymes. DNA damage by ROS activates poly(ADP-ribose) polymerase 1 (PARP1), which adds ADP-ribose polymers (PARs) to the mitochondrial base excision repair (BER) enzymes, exo/endonuclease G (EXOG) and DNA polymerase gamma (Polγ), and causes mitochondrial DNA repair [71,72].

When HUVECs are cultured on polysulfone support covered with fibronectin, they are firmly attached to the porous membrane surface. The detachment of cells at the end of the experiment is difficult; many of them cannot be detached or are destroyed during this procedure. Therefore, it is difficult to reliably estimate the number of cells in the culture. We used the amount of lactate produced by HUVECs as a comparative indicator of the number of cells after 7 and 14 days of culturing. We could only apply this method to HUVECs cultured without HUASMCs, due to the fact that HUVECs and HUASMCs were co-cultured on two membranes in one culture well and were releasing lactate into the same medium. It was shown [73] that lactate production depends on the number of HUVECs cultured in the culture flasks in medium with normal glucose concentration and that it increases with increasing density of cells in the culture. Moreover, it was demonstrated that the proliferation index of cells (determined as the sum of the cells in all generations divided by the number of original parent cells) cultured in flasks increased with culture duration. Although, in our tests, no significant effect of glucose concentration on the apoptosis of HUVECs after 7 days of culturing has been demonstrated, lactate production was the same for cultures with high and variable glucose concentrations and 2.4 times lower than in cultures in medium with normal glucose concentration. We presume that the number of HUVECs in cultures in medium with high and variable glucose concentrations was probably close to the same and 2.4 times lower than the number of cells cultured in medium with normal glucose concentration. Because of the insignificant differences in percentage of apoptotic cells, we conclude that it was due to the slower proliferation of HUVECs cultured in medium with high and variable glucose concentrations compared to the proliferation of cells cultured in medium with normal glucose concentration. After 14 days of culturing, the apoptosis of HUVECs cultured in medium with variable glucose concentration was significantly higher than that observed with high glucose concentration. At the same time, the production of lactate by these cells was 1.1 times lower than the production of lactate by cells cultured in medium with high glucose concentration. There was also no significant difference between normal and high glucose concentrations in terms of the percentage of apoptotic HUVECs. HUVECs cultured in medium with high and variable glucose concentrations produced about 4.5 times less lactate compared to the cells in medium with normal glucose concentration. After 14 days, the lower number of HUVECs in medium with variable glucose levels than that in high glucose levels can be explained not only by slower proliferation (as after 7 days of the culture) but, additionally, by a higher apoptosis rate.

## 4. Materials and Methods

### 4.1. HUVEC and HUASMC Isolation

HUVECs were isolated from umbilical cords obtained by Caesarean section using the procedure described by Jaffe et al. [74]. After the first passage, cells were frozen at liquid nitrogen temperature (−196 °C) and stored until use for target tests. The cultures with HUVECs were conducted in the medium M199 (Sigma Aldrich, St. Louis, MO, USA) enriched with 20% FBS (Gibco, Paisley, UK), 10 µg/mL endothelial cell growth factor ECGS (Merck Millipore, Darmstadt, Germany), 15 IE/mL heparin (Polfa S.A., Warsaw, Poland), and 100 µmol/L penicillin with streptomycin (Sigma Aldrich, St. Louis, MO, USA). HUASMCs were isolated from umbilical arteries of the same umbilical cords which were used to isolate HUVECs according to a modified method described by Ulrich-Merzenich et al. [75]. DMEM (1X) medium (Gibco, Paisley, UK) containing 4.5 g/L of glucose, 15 IU of heparin (Polfa S.A., Warsaw, Poland), and 100 IU of penicillin with streptomycin (Sigma Aldrich, St. Louis, MO, USA) was used to culture HUASMCs. Cultures of both types of cells were performed in an incubator in controlled atmosphere (5% CO_2_ and 95% air) at 37 °C. In the target tests, we used cells (both HUVECs and HUASMCs) after the second passage. A mixture of cells originating from 10 different donors was used to reduce the variability of results caused by inter-subject differences.

We obtained informed written consent from all patients whose umbilical cords were used. The study was approved by the Bio-Ethical Committee of the Medical University of Warsaw (decision no. KB/12/2017), and it conformed with the provisions of the Declaration of Helsinki.

### 4.2. HUVEC and HUASMC Cultures with Different Glucose Levels

**Figure 8:**
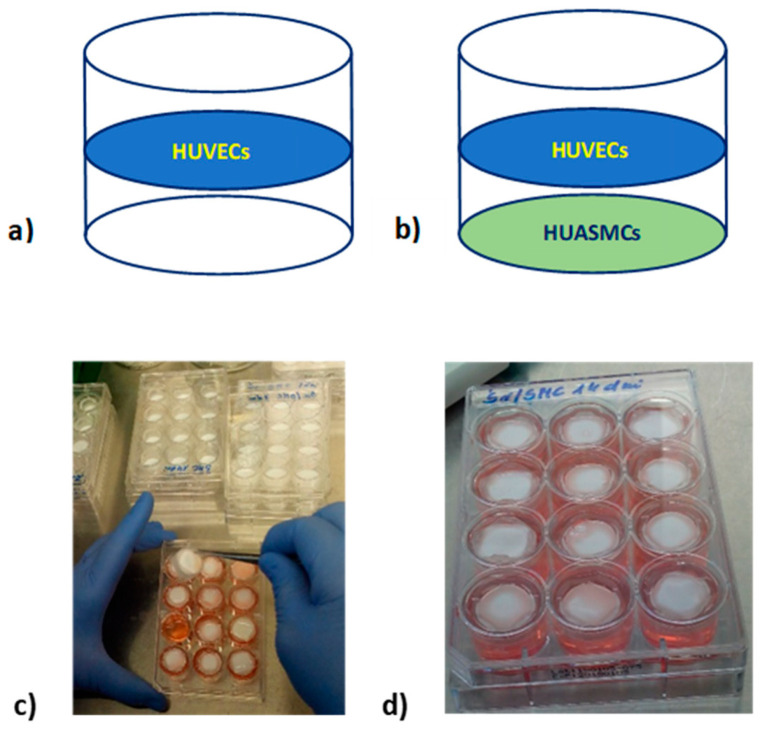
Culture of HUVECs seeded on the PSU membrane modified with fibronectin (**a**); co-culture of HUVECs and HUASMCs (HUVECs/HUASMCs) seeded on two flat membranes modified with fibronectin immobilized in the one insert (**b**); establishing a cell culture in a 12-well culture plate (**c**); 12-well culture plate with inserts with membranes and seeded cells (**d**).

Cultures were performed on 2 cm^2^ polysulfone flat semi-permeable membranes which were immobilized in self-made inserts. Such membranes allow for separate seeding of different types of adherent cells (separating them from each other) while allowing the exchange of signaling molecules between cells in a manner similar to that taking place in blood vessels. Membranes were modified with fibronectin to allow cell adhesion. HUVECs were seeded with a density of 4.6 × 10^3^/cm^2^ and HUASMCs with a density of 3.5 × 10^3^/cm^2^. Inserts with membranes with seeded cells were placed in 12-well culture plates. The wells were filled with culture medium with the appropriate glucose concentration (Figure 8).

The following experiments were carried out:

- Culture of HUVECs seeded on the PSU membrane modified with fibronectin (Figure 8a).
- Co-culture of HUVECs and HUASMCs (HUVECs/HUASMCs) seeded on two flat membranes, located close to each other, modified with fibronectin and immobilized in the one insert (Figure 8b).

Glucose levels in the culture medium were as follows: normal—5 mmol/L (N), high—20 mmol/L (H), variable with a daily change from N to H (H/N) and vice versa. Cultures were carried out for 7 and 14 days. The culture medium was replaced once every 24 h in all cultures independent of whether the glucose concentration was constant or variable. Twelve replicates of the cultures were performed for each glucose concentration in the culture medium (normal, high, and variable) and for each culture duration (7 and 14 days), i.e., a total of 144 insert cultures on 12 12-well culture plates. After 7 and 14 days of culture, cells were harvested separately from each membrane using Tripsin-EDTA solution (Sigma Aldrich, St. Louis, MO, USA) and counted in a Bürker Counting Chamber. For analyses, cells detached from one membrane were used, or, if it turned out that there were too few cells grown in the same conditions, cells detached from several membranes were pooled. Therefore, each analysis was performed with 12 replications or less.

### 4.3. Apoptosis Analysis

An Annexin V-FITC/7AAD apoptosis detection kit (BD Pharmingen, San Diego, CA, USA) was used to measure the early apoptosis and late apoptosis of HUVECs. The total percentage of HUVECs in apoptosis was calculated. After 7 and 14 days of culture, cells were harvested using Tripsin-EDTA solution (Sigma Aldrich, St. Louis, MO, USA), washed in PBS, suspended in a binding buffer in tubes for Annexin-FITC and 7-AAD, and kept at 37 °C for 20 min under dark conditions as per the manufacturer’s instructions. The final concentration of cells was 1 × 10^5^ cells/mL. Samples stained with Annexin V and 7AAD were quantitatively analyzed at an emission wavelength of 488 nm and an excitation wavelength of 570 nm by flow cytometry (Becton Dickinson, San Jose, CA, USA). As a result, the percentages of cells in early apoptosis (Annexin V-positive, 7AAD-negative) and late apoptosis (Annexin V-positive, 7AAD-positive) were obtained. Percentages of cells in early and late apoptosis were calculated as mean values of the percentage of early and late apoptotic cells in each pool of cells detached from membranes. The percentage of cells in apoptosis was calculated as the mean value of the sums of the percentage of early and late apoptotic cells in each pool of cells detached from membranes.

### 4.4. Mitochondrial Membrane Potential Analysis

The fluorescent probe JC-1 (Invitrogen by Thermo Fisher Scientific, Eugene, OR, USA) was used to determine the mitochondrial membrane potential of HUVECs. After 7 and 14 days of culture, cells were harvested, washed in PBS, and incubated with JC-1 for 15 min at 37 °C as per the manufacturer’s instructions. The final concentration of cells was 1 × 10^5^ cells/mL. The cells were washed with PBS, and cell staining was determined by flow cytometry (Becton Dickinson, San Jose, CA, USA).

### 4.5. Intracellular Reactive Oxygen Species (ROS) Analysis

ROS levels were determined using the DCFH-DA assay (Invitrogen by Thermo Fisher Scientific, Eugene, USA). This fluorogenic dye measures hydroxyl, peroxyl, and other reactive oxygen species activity within the cell. After 7 and 14 days of culture, cells were harvested, washed in PBS, and incubated with DCFH-DA for 20 min at 37 °C as per the manufacturer’s instructions. The final concentration of cells was 1 × 10^5^ cells/mL. The cells were washed with PBS, and flow cytometry (Becton Dickinson, San Jose, CA, USA) was used for the estimation of ROS accumulation.

### 4.6. Lactate Production Analysis

Samples for measuring daily lactate production by HUVECs were taken once daily before changing the culture medium in the culture wells. Lactate measurements were performed on a YSI 2300 Stat Plus Analyzer (Yellow Springs Instruments, Yellow Springs, OH, USA). The obtained results of the analyses were presented as the daily lactate production and as the total lactate production by HUVECs in the 14-day culture period. For each time plot of total lactate production, a trend line was calculated for which R^2^ had the maximum value. The total lactate production after 7 and 14 days of HUVECs cultured without HUASMCs was calculated. Measurements of lactate production by HUVECs co-cultured with HUASMCs were not possible, because both types of cell were co-cultured in one insert in common culture medium.

### 4.7. Statistical Analysis

The results for apoptosis, mitochondrial potential, ROS production, and lactate production obtained in HUVECs cultured for 7 and 14 days were compared. The results are expressed as mean ± SEM (standard error of mean). The percentages of apoptotic cells in the cultures, the values of mitochondrial potential, ROS production, and lactate production are presented as absolute values calculated separately for HUVECs cultured alone and co-cultured with HUASMCs after 7 and 14 days. The normality of distribution of the analyzed variables was verified using the Shapiro–Wilk test, or, if the number of measurement points was too small, a normal distribution was assumed. Statistical analysis was performed using Statistica ver. 10 (StatSoft Inc., Tulsa, OK, USA). A three-way analysis of variance (ANOVA) was used to analyze HUVECs viability (percentage of the early apoptotic or late apoptotic cells or both), mitochondrial potential, ROS production, glucose consumption, or lactate production. The following three independent factors were analyzed: glucose concentration in the culture medium (N, H, H/N), culture duration (7 and 14 days), and the type of culture (HUVECs alone or in co-culture with HUASMCs). The interactions between these three independent factors as well as interactions of these factors in pairs were considered. ANOVA was followed by a Bonferroni post hoc test to identify differences between groups. A value of *p* < 0.05 was considered to indicate statistical significance.

## 5. Conclusions

The results of our study confirm that fluctuations in glucose concentration in culture medium, mimicking glucose fluctuations in people with diabetes, are more detrimental to the viability of endothelial cells than a constant high concentration of glucose. High and variable glucose concentrations in the culture medium slow down HUVECs proliferation compared to the culture carried out in medium with normal glucose concentration. The results of the study indicate that smooth muscle cells co-cultured with endothelial cells on a semi-permeable polysulfone membrane affect the viability of the endothelial cells in a manner dependent on the concentration of glucose in the culture medium and the duration of the culture.

## Data Availability

The data presented in this study are available on request from the corresponding author.
